# Comparison of the Effect of Anesthesia With Midazolam-Fentanyl Versus Propofol-Remifentanil on Bispectral Index in Patients Undergoing Coronary Artery Bypass Graft

**DOI:** 10.5539/gjhs.v7n5p233

**Published:** 2015-03-16

**Authors:** Naser Hemmati, Abdol Hamid Zokaei

**Affiliations:** 1 School of Medicine, Kermanshah University of Medical Sciences, Kermanshah, Iran

**Keywords:** Remifentanil, propofol, midazolam, fentanyl, bispectral index, coronary artery bypass grafting

## Abstract

The aim of this study was to compare the effect of anesthesia with midazolam-fentanyl versus propofol-remifentanil on the BIS (bispectral index) in patients undergoing coronary artery bypass grafting (CABG). Sixty-four patients undergoing CABG were randomly assigned to one of two study groups: midazolam-fentanyl (MF, N= 32) or propofol-remifentanil (PR, N= 32). The BIS was measured before induction of anesthesia, five minutes after induction of anesthesia, at skin incision, sternotomy, pericardiotomy, aorta cannulation, onset of cardiopulmonary bypass, during rewarming, five minutes after separation from cardiopulmonary bypass, at thorax closure, and at the end of the surgery. There were no significant differences between the two groups with regard to age and gender. The difference in mean BIS between the two groups was significant (*P* < 0.05) at all times, except before induction, five minutes after induction, at skin incision and on rewarming. Changes in the BIS were lower in the PR group than in the MF group. Both techniques can provide adequate anesthesia in patients undergoing CABG. However, the probability of awareness during anesthesia is lower with propofol-remifentanil than with midazolam-fentanyl.

## 1. Introduction

In cardiac surgery, anesthetic agents and techniques used for anesthesia should be selected according to the cardiac pathophysiology of the patient and other comorbid conditions (Nussmeier et al., 2005). Cardiac anesthesia, including high dose opioids (e.g., fentanyl and sufentanil) and low dose intravenous anesthetic agents is commonly used to achieve intraoperative hemodynamic stability and effective attenuation of sympathetic responses to surgical stimuli (Ruggeri et al., 2011) without causing severe myocardial depression (Howie et al., 2001; Rauf et al., 2005; Steinlechner et al., 2007). However, high doses of opioids do not suppress all responses to surgical stimuli, and adjusting the level of anesthesia to prevent various degrees of surgical stimulation remains a problem when using traditional opioids (Howie et al., 2001).

Anesthesia using propofol and remifentanil has been suggested as a safe anesthetic technique in cardiac surgery (Ouattara et al., 2003). Remifentanil has a potent affinity for the mu opioid receptor, with a half-life of less than 10 minutes (Hogue et al., 1996; Michelsen et al., 2001; Steinlechner et al., 2005). Use of remifentanil in conjunction with propofol (as a sedative hypnotic agent) may provide deep suppression of responses to surgical stimuli (Howie et al., 2001).

The measurement of depth of anesthesia is of clinical interest for titrating anesthetic drugs and for avoiding patient awareness (explicit recall of sensory perceptions) during general anesthesia (Schmidt et al., 2003). The bispectral index (BIS) monitor, one of the first anesthesia depth monitors approved by the US Food and Drug Administration (Weil etal., 2008; Kreuer etal., 2005; Lysakowski et al., 2001; Schmidt et al., 2002; Nasraway et al., 2002; Avidan et al., 2008; Akcalki et al., 2008) has been widely accepted in clinical practice (Avidan et al., 2008).

The objective of this study was to compare the effect of anesthesia using midazolam-fentanyl vs. propofol-remifentanil on the BIS values in patients undergoing coronary artery bypass graft surgery.

## 2. Methods

This study was a randomized clinical trial. After obtaining approval from the institutional ethics committee of Kermanshah University School of Medicine, Kermanshah, Iran informed consents were obtained from 64 patients undergoing elective coronary artery bypass graft surgery on cardiopulmonary bypass.

Inclusion criteria were American Society of Anesthesiologists physical status1or 2 and no evidence of heart failure at preoperative clinical assessment. The sampling method was of convenient method and considering a minimum difference of 7 between the two groups, the sample size in each group was calculated using the following formula:


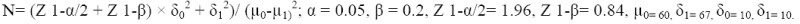


The patients were randomly assigned to one of two study groups: midazolam–fentanyl (MF, N= 32) or propofol-remifentanil (PR, N = 32). None of the patients were lost to follow.

Exclusion criteria included an ejection fraction less than 30%, a report of dyskinetic or akinetic regions or ventricular aneurysm on echocardiography or angiography, left ventricular end diastolic pressure more than 18 mmHg on angiography, and severe reduction in blood pressure after induction needing intervention. Patients with a history of sternotomy, prolonged use of benzodiazepines or opioids were also excluded.

On the morning of surgery, patients received the usual medications and were premedicated with morphine sulfate 0.1 mg/kg and promethazine 0.5 mg/kg intramuscularly one hour before transfer to the operating room. At the time of entry to the operating room, electrocardiographic monitoring, arterial cannulation and central venous catheter insertion were done. BIS values were monitored using the Aspect Medical Systems A-2000 BIS monitor (Leiden, Netherlands, version 2.20). The BIS monitor used a standard disposable sensor (BIS™ sensor XP, Aspect Medical Systems, Newton, MA) that was applied to the patient’s forehead, as recommended by the manufacturer.

All patients were oxygenated with pure100% oxygen for at least three minutes and received 5-10 mL/kg normal saline. In the MF group, induction was initiated with fentanyl (7.5 μg/kg) and midazolam (0.15 mg/kg) and maintained with midazolam (1 μg/kg/min) and fentanyl (4 μg/kg/hour). Before skin incision and sternotomy, fentanyl 2 μg/kg was given. In the PR group, induction was initiated with remifentanil 1 μg/kg and propofol 0.2 μg/kg, and maintained with propofol 100 μg/kg/min and remifentanil 0.5 μg/kg/min. Musculoskeletal relaxation for intubation were achieved using pancuronium 0.1 mg/kg and continued using cisatracurium 5 μg/kg/hour in both groups. Other usual cardiac medications and procedures were equal in the two groups. BIS values before induction, five minutes after induction, at skin incision, sternotomy, pericardiotomy, aorta cannulation, start of cardiopulmonary bypass, during rewarming, five minutes after separation from cardiopulmonary bypass, at thorax closure, and at the end of the surgery (skin closure) were measured. If BIS ≥ 50 was observed, an additional dose of propofol (30 mg) in the PR group and midazolam (5 mg) in Mf group was injected as a bolus and repeated at five-minute intervals until BIS reached less than 50.

Statistical calculations were performed using the SPSS software for Windows version14.0 (SPSS Inc, Chicago, IL). Statistical analysis was performed using the independent *t*-test or Wilcoxon signed-rank test for comparing quantitative variables. Qualitative variables were compared using the Chi-squared test or Fisher’s exact test. Statistical significance was defined as *P* < 0.05. Data are presented as the mean ± standard deviation (SD).

The Ethics Committee of our medical university approved the protocol of the study. All details of the study were explained to the patients and if agreed, written informed consent was obtained from them.

## 3. Results

Of the 64 patients enrolled in the study, 45 were male. Mean (±SD) age was 54.5 (± 8.1) years. There was no significant difference between the two groups with regard to age (Table 1).

**Table 1:** Comparison of mean (±SD) age between the two groups

Group	Number of patients	Mean (±SD) age	T	*P* value
MF	32	53.22 (±7.13)	1.14	0.26
PR	32	55.56 (±8.64)		

Abbreviations: MF: midazolam-fentanyl; PR: propofol-remifentanil; SD: standard deviation.

Comparison of the two groups with regard to gender using the Fisher’s Exact test and 95% confidence interval (CI) did not show any significant difference (*P =* 0.37). Differences in the mean BIS values between the two groups using the independent *t*-test were statistically significant at all times, except before induction, five minutes after induction, when skin incision was done, and during rewarming (Table 2).

**Table 2:** Comparison of mean (BIS) values at different times of anesthesia between the MF and PR groups

Time	Group	Mean	SD	t	P value
Pre-induction	MF	90.74	9.56	0.168	0.868
	PR	90.38	6.02		
Five minutes after induction	MF	53.96	8.09	-1.408	0.165
	PR	57.28	9.72		
After incision	MF	50.93	8.94	1.173	0.093
	PR	47.09	8.08		
After sternotomy	MF	53.89	9.07	5.642	0.009
	PR	43.16	5.3		
After pericardiotomy	MF	49.56	10.28	5.266	0.009
	PR	38.59	5.29		
After aorta cannulation	MF	49.89	9.48	2.023	0.047
	PR	44.84	9.49		
At cardiopulmonary bypass onset	MF	55.26	16.49	3.533	0.001
	PR	43.03	9.71		
Rewarming	MF	46.7	12.6	1.793	0.078
	PR	40.81	12.53		
Five minutes after end of cardiopulmonary bypass	MF	49.22	12.39	3.707	0.001
	PR	38.47	9.34		
After thorax closure	MF	54.13	14.2	4.902	0.0009
	PR	40.03	6.81		
Surgery end	MF	53.04	12.93	4.600	0.009
	PR	41.16	5.95		

Abbreviations: BIS: bispectral index; MF: midazolam-fentanyl; PR: propofol-remifentanil; SD: standard deviation.

## 4. Discussion

Several brain function monitors based on the electroencephalogram have been used to assess depth of anesthesia. The most widely used is the BIS monitor, which has been approved by the US Food and Drug Administration (Avidan et al., 2008; Leslie et al., 1996; Kearse et al., 1998). The BIS monitor processes a single frontal electroencephalographic signal to calculate a dimensionless number that provides a measure of the patient’s level of consciousness. BIS values range from 100 to 0, reflecting the awake state and the absence of brain activity, respectively. BIS values between 40 and 60 indicate adequate general anesthesia for surgery, and values below 40 indicate a deep hypnotic state (Avidan et al., 2008).

In this study, the effect of two different anesthetic techniques, ie, MF and PR, on the BIS index were compared in patients undergoing coronary artery bypass surgery. The results of our study showed a difference in BIS values between the two groups that was significant at all times during anesthesia except at preinduction, five minutes after induction, after skin incision, and during rewarming, whereas BIS values in the PR group was lower than in the MF group at all times during anesthesia.

In a similar study, Lehmann et al investigated the effects of two anesthetic regimens (sufentanil- midazolam versus propofol-remifentanil) on the BIS index in patients undergoing aortocoronary bypass graft surgery. In that study, BIS values in the PR group was lower than in the midazolam-sufentanil group, but no BIS value indicating awareness during anesthesia was observed in either group (Lehmann et al., 2000).

9=Intravenous anesthesia based on administration of propofol accompanied by a narcotic is a common anesthetic technique, in which the compositions of anesthesia can be changed (Billard et al., 1997). Many investigators have shown that there is an appropriate correlation between BIS and blood concentration of propofol (Glass et al., 1997; Malinge et al., 1998; Doi et al., 1997). On the other hand, the majority of anesthetists believe that the anesthetic effects of propofol can be increased by adding an opioid (Glass et al., 1997; Malinge et al., 1998; Doi et al., 1997; Vuyk et al., 1995). Fentanyl is a synthetic opioid and has a powerful affinity for the mu receptor. This drug has strong analgesic and hypnosedative effects. However, because of its pharmacokinetic characteristics, this opioid may accumulate in the body, causing prolonged recovery and respiratory depression (Lison et al., 2007). Remifentanil is a short- acting opioid which also has a strong affinity for the mu receptor and has unique pharmacokinetic properties, with an elimination half-life of approximately 10–20 minutes and a context- sensitive half- life of 3–4 minutes, regardless of the duration of infusion (Ruggeri et al., 2011; Hogue et al., 1996). This drug has been administered in high risk cardiac patient successfully (Lehmann et al., 1999). Rapid recovery from anesthesia has been noted, even with prolonged infusion of remifentanil (Ruggeri et al., 2011). The aims of so-called fast-track cardiac anesthesia include early tracheal extubation and decreased length of intensive care unit and hospital stay, with subsequent cost reduction. Any intervention that reduces postoperative complications, and therefore total length of hospital stay, should be considered as an integral component of fast-track cardiac anesthesia. Many authors have demonstrated that a reduction in opioid dosage is a key component of fast- track cardiac anesthesia, and immediate onset and offset of the analgesic effect of remifentanil makes it a good agent to control painful stimuli during cardiac surgery and rapid recovery from anesthesia (Myles et al., 2005). On the basis of these properties, remifentanil can be administered as a suitable anesthetic in patients undergoing fast-track coronary artery bypass graft surgery.

We faced limitation in this study including additional effects of the anesthetic agents and lack of survey for any side effects of the anesthetics. Also, except for age and gender, no other baseline characteristics were recorded.

## 5. Conclusion

Both anesthesia techniques studied herein can provide adequate anesthesia in patients undergoing coronary artery bypass grafting. However, the probability of awareness during anesthesia is lower with propofol-remifentanil than with midazolam-fentanyl.
